# Patient-Reported Outcome Measures of Flapless Corticotomy with Low-Level Laser Therapy in En Masse Retraction of Upper Anterior Teeth: A Three-Arm Randomized Controlled Trial

**DOI:** 10.3390/clinpract13060132

**Published:** 2023-11-22

**Authors:** Mudar M. Mousa, Mohammad Y. Hajeer, Ahmad S. Burhan, Khaldoun M. A. Darwich, Wael H. Almahdi, Ossama Aljabban, Mohammed A. Awawdeh, Imad Addin Almasri

**Affiliations:** 1 Department of Orthodontics, Faculty of Dentistry, University of Damascus, Damascus P.O. Box 30621, Syria; mudar.mousa@damascusuniversity.edu.sy (M.M.M.); prof.ahmadburhan@damascusuniversity.edu.sy (A.S.B.); 2 Department of Oral and Maxillofacial Surgery, Faculty of Dentistry, University of Damascus, Damascus P.O. Box 30621, Syria; khaldoun.darwich@damascusuniversity.edu.sy; 3 Department of Periodontics, Faculty of Dentistry, University of Damascus, Damascus P.O. Box 30621, Syria; wael.almahdi@damascusuniversity.edu.sy; 4 Department of Restorative Dentistry and Endodontics, Faculty of Dentistry, Damascus University, Damascus P.O. Box 30621, Syria; ossama.jabban@damascusuniversity.edu.sy; 5 Preventive Dental Science Department, College of Dentistry, King Saud bin Abdulaziz University for Health Sciences (KSAU-HS), Riyadh 11426, Saudi Arabia; awawdehm@ksau-hs.edu.sa; 6 Department of Applied Sciences, Faculty of Economics, Damascus University, Damascus P.O. Box 30621, Syria; imad_almasri27@yahoo.com

**Keywords:** en masse retraction, peizocision, LLLT, pain, discomfort, mini-implants

## Abstract

(1) Background: This study aimed to compare patient-reported outcome measures when accelerating en masse retraction between the piezocision procedure and the subsequent application of low-level laser therapy (FC+LLLT), with the piezocision alone (FC), and in a control group. (2) Methods: A three-arm randomized controlled trial (RCT) was conducted involving 60 patients (41 females and 19 males) with Class II division I malocclusion. The en masse retraction was performed using NiTi closed coil springs attached to miniscrews. The LLLT was performed using an 808 nm Ga-Al-As diode laser. Patient responses regarding pain, discomfort, swelling, and chewing difficulties were reported at ten assessment points. (3) Results: The greatest pain levels were observed 24 h after the application of force during the first and third months of retraction. The mean pain, discomfort, swelling, and chewing difficulties were significantly smaller in the control group than in the FC and FC+LLLT groups. High satisfaction levels were reported in all three groups (*p* < 0.05). (4) Conclusions: The accelerated en masse retraction via piezocision, followed by a small course of LLLT, was accompanied by significantly fewer pain, discomfort, and chewing difficulties than the control group. LLLT is a valuable addition to piezocision, with an improved patient experience.

## 1. Introduction

The duration of orthodontic treatment presents a significant challenge for both orthodontists and patients, and even deters some patients from undergoing treatment [1]. Shortening the duration of orthodontic treatment is a primary goal for both orthodontists and patients, especially adult patients [2], as prolonged treatment can lead to adverse effects such as root resorption, periodontal disease, and dental caries [3,4]. Furthermore, patients often experience discomfort and pain associated with various stages of therapy, with pain being the most disliked aspect of orthodontic treatment [5].

Several methods have emerged to improve orthodontic tooth movement and shorten the duration of orthodontic treatment, with surgical methods being the most popular, including traditional corticotomy [6,7], and interseptal alveolar surgery. However, these techniques have shown low acceptance among patients due to their invasive nature and postoperative complications.

Minimally invasive techniques, such as piezocision [8,9], micro-osteoperforations (MOPs) [10], corticision [11], and laser-assisted flapless corticotomy [12], have gained popularity due to their ability to minimize postoperative discomfort and reduce recovery time. On the other hand, non-surgical interventions like low-level laser therapy (LLLT) [13,14], light vibrational forces [15], light-emitting diodes [16], and pulsed electromagnetic waves [17] are currently widely used.

LLLT has been shown to be effective in accelerating tooth movement and reducing pain, as supported by recent studies [14,18]. Consequently, there is a growing interest in using LLLT as an adjunct to traditional orthodontic treatment to take advantage of the acceleration properties of minimally invasive methods and the analgesic and acceleration properties of LLLT [19,20].

Reviewing the literature reveals some recent research work combining two or more acceleration methods to accelerate the orthodontic movement, such as combining self-ligating brackets with one or more acceleration methods [21], or the use of LLLT with minimally invasive methods like corticotomy [22], MOPs [23], and piezocision for canine retraction [24]. A recent systematic review showed that there were several published studies about the levels of pain and discomfort when using surgically assisted acceleration methods [25]. However, no studies have examined patient-reported outcome measures associated with the combination of piezocision with LLLT in the context of the en masse retraction of upper anterior teeth. Therefore, the aim of the current RCT was to assess the patient-reported outcome measures when accelerating the en masse retraction of upper anterior teeth in adult patients using piezocision combined with the subsequent application of LLLT compared to piezocision alone, or with no acceleration method.

The null hypothesis (H0) stated that there were no significant differences between the piezocision plus subsequent LLLT application, piezocision alone, and traditional en masse retraction groups in terms of the levels of pain, discomfort, swelling sensation, chewing difficulties, satisfaction, and acceptance at all assessment times.

The research hypothesis (H1) stated that there were significant differences between piezocision with subsequent LLLT application, piezocision alone, and traditional en masse retraction groups in the levels of pain, discomfort, swelling sensation, chewing difficulties, satisfaction, and acceptance at all assessment times

## 2. Materials and Methods

### 2.1. Study Design and Setting

This is a three-arm, parallel-group, randomized controlled trial (RCT) conducted between February 2021 and December 2022, involving individuals enrolled in the Department of Orthodontics at Damascus University’s Faculty of Dentistry. The Consolidated Standards of Reporting Trials (CONSORT) guidelines were followed to optimize the writing of the study [26]. The Local Research Ethics Committee of the University of Damascus approved the study (UDDS-632-15012021/SRC-1679), which is registered in the Clinical Trials database (ID: NCT05656898). The research was supported by Damascus University’s Postgraduate Research Budget (Ref no. 23654267DEJ).

### 2.2. Sample Size Calculation

To determine the sample size, Minitab^®^ (version 18, State College, PA, USA) was used. It was assumed that the minimum clinically important difference in pain perception on the visual analog scale (VAS) requiring detection was 15 mm. The variability of this parameter (i.e., the SD) was estimated from a previous study as being 12.26 mm out of 100 mm [27]. As a result, a minimum of 18 participants per group was required when using a one-way ANOVA with 90% statistical power and a 5% significance level. Taking into account any potential dropout (not exceeding 10%), a total of 20 patients were required for each group.

### 2.3. Patient Recruitment and Entry in This Trial

#### Inclusion/Exclusion Criteria

Participants in this study were selected based on the following criteria: (1) healthy patients aged between 17 and 28 years from both genders; (2) Class II division I malocclusion with an indication for the extraction of the upper first premolars, which includes an overjet of 4–10 mm and normal or long face growth patterns; (3) skeletal Class II relationship (4 < ANB ≤ 7); (4) a normal overbite, which is more than 0 mm and less than 4 mm; (5) good oral hygiene, as evidenced by a probing depth of ≤3 mm and no alveolar bone loss assessed radiographically; and (6) patients with no missing teeth (except for third molars).

The exclusion criteria for the study were: (1) previous orthodontic treatment; (2) systemic disease or medication that affects orthodontic movement; (3) absence of one of the maxillary teeth (except third molars); (4) poor oral hygiene or existing periodontal disease, which was judged by probing depth ≥ 4 mm, radiographic evidence of bone loss, gingival index >1, and plaque index > 1; and (5) moderate to severe crowding on the maxillary teeth (Little’s Irregularity Index ≥ 4)

**Figure 1:**
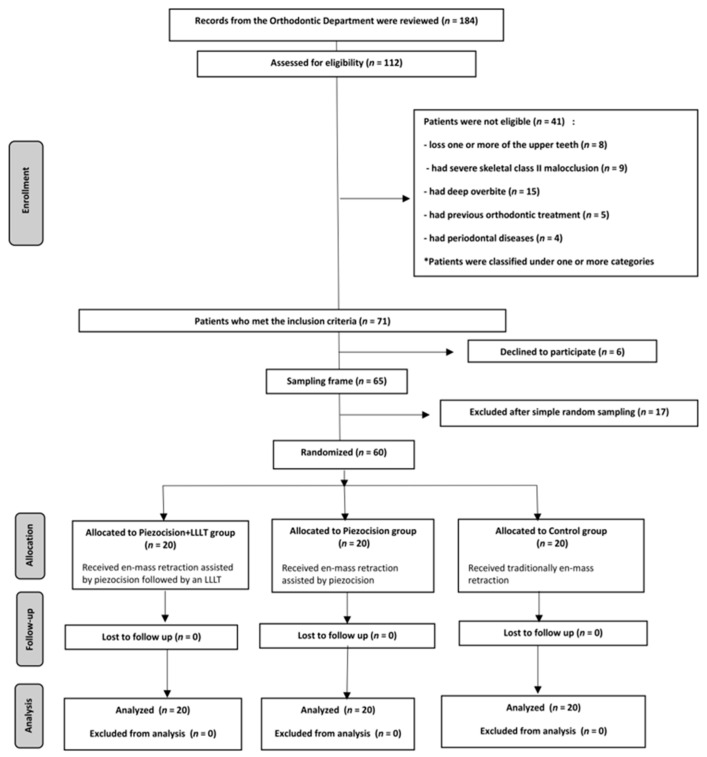
CONSORT flow diagram of patient recruitment, assignment, follow up, and entry into data analysis. The LLLT included the use of Ga-Al-As diode laser with a wavelength of 808 nm, a power of 1.1 watts, an energy of 1864 joules/point, and an application of 15 s per point.

A total of 112 patients attending the Department of Orthodontics at Damascus University’s Faculty of Dentistry were clinically examined, and 71 patients met the inclusion criteria. Then, the candidate patients were informed of the research details, and a detailed explanation of the orthodontic, surgical, and LLLT procedures intended for this study was provided. As a result, 65 patients agreed to participate in this research. Later, 60 of the 65 patients were chosen randomly (Figure 1). All selected patients were given written information sheets to read before signing consent forms.

### 2.4. Randomization, Allocation Concealment, and Blinding

Simple randomization was carried out to assign the 60 patients into three groups: (1) en masse retraction assisted by flapless corticotomy combined with the later application of LLLT (FC+LLLT), (2) en masse retraction assisted by flapless corticotomy (FC), and (3) conventional en masse retraction (CONT). An academic member who was not involved in this research created a list of random numbers using Minitab^®^ Version 19.1 (Minitab Inc., State College, PA, USA), with an allocation sequence of 1:1:1 ratio. At the end of the leveling and alignment stage, the sequentially numbered, sealed, and opaque envelopes containing the allocation sequence were opened. Blinding was applied only to the outcomes’ assessor, and it did not apply to either the patient or principal researcher (M.M.M). 

### 2.5. Interventions

#### 2.5.1. Leveling and Alignment Phase

The principal investigator (M.M.M) performed the orthodontic treatment under the supervision of one of the coauthors (M.Y.H) at the Orthodontic Department of the Faculty of Dentistry of Damascus University. At the beginning of the treatment, pre-adjusted fixed orthodontic appliances; MBT 0.022-inch slot size (Votion™, OrthoTechnology^®^, Tampa, FL, USA), were bonded to all patients. Then, self-drilling titanium temporary anchorage devices with 1.6 mm diameter and 8 mm length (3S screw, Hubit^®^, Seoul, Republic of Korea) were inserted bilaterally at the gingival–mucosal junction approximately 8–10 mm high above the archwires between the maxillary second premolar and the 1st molar, and these teeth were attached to the mini-screw with a ligature wire. Thereafter, patients were referred for extraction of the maxillary 1st premolars. The archwires were applied according to the following sequence: the initial wire 0,014-in Nitinol (NiTi), followed by 0.016-in NiTi, 0.016 × 0.022-in NiTi, 0.017 × 0.025-in NiTi, 0.019 × 0.025-in NiTi, and finally the last wire 0.019 × 0.025-in stainless steel. However, the last wire was kept for two weeks to become neutral before starting the process of en masse retraction. After that, the used wires were replaced with wires of the same size, with soldered hooks placed distally to the lateral incisors.

#### 2.5.2. The Flapless Corticotomy Procedure in the FC+LLLT and FC Groups

**Figure 2:**
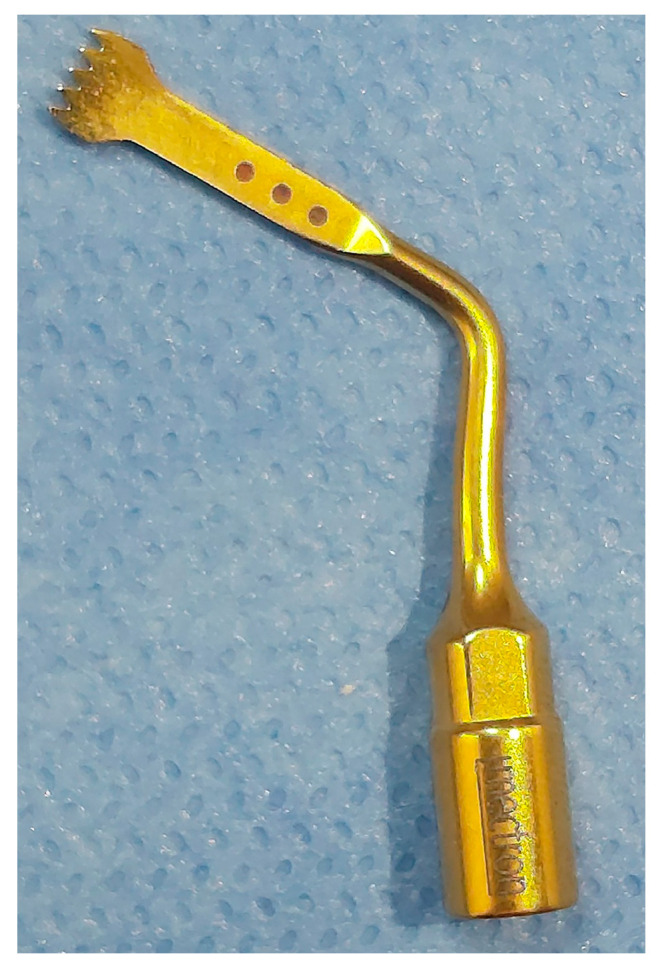
BS1 cutting tip used for performing the cortical cuts.

**Figure 3:**
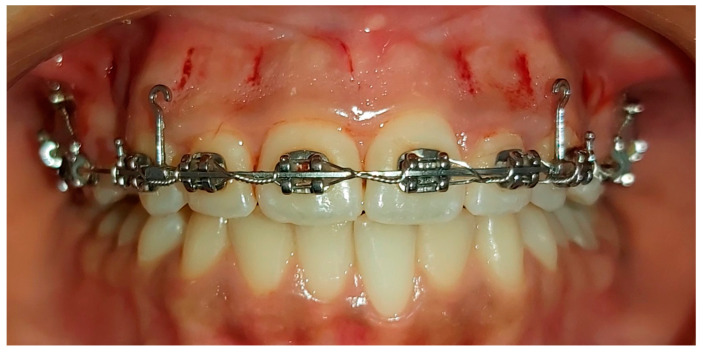
The minimally invasive piezocision intervention.

The patients received the same aforementioned procedures with the CONT group, and 4 days before the start of the en masse retraction, piezosurgery was performed by the same principal investigator (M.M.M) under the supervision of one of the coauthors (W.A.H) at the Oral and Maxillofacial Surgery Department of the same university. Before the surgical intervention, the patients were asked to use chlorhexidine 0.12% rinse for 60 s. On the buccal aspect, two vertical incisions were made distal to the canine at the site of extraction of the 1st premolars at both sides. Moreover, one vertical gingival incision was made between each of the roots of two adjacent teeth in the anterior region using blade No. (15). However, the same incisions were also performed on the palatal aspect. So, the number of incisions was 18 in total (9 from the buccal side and 9 from the palatal one). These incisions started at a distance of 5 mm apically from the interdental papilla and were 5 mm in length (The purpose of these incisions is to penetrate the periosteum in order to allow the piezosurgery cutting tip to enter and reach the cortical bone), then a piezosurgery micro saw with a BS1 cutting tip (Piezosurgery^®^, Mectron, Carasco, Italy; Figure 2) was inserted to create alveolar cortical cuts with 3 mm depth (Figure 3). No sutures were applied after performing the incisions. Finally, the area was washed well with physiological serum (sodium chloride) at a concentration of 0.9%. Then, the surgical site was covered using a piece of gauze impregnated with Idoform (BIOGAR Povidone, BIOGAR©, Lattakia, Syria) that was applied for an hour. All patients were strictly asked to adhere to good oral hygiene and avoid nonsteroidal anti-inflammatory drugs using. Paracetamol (acetaminophen, 500 mg) was allowed only when needed, provided the questionnaires had first been answered.

#### 2.5.3. The LLLT Procedure in the FC+LLLT Group

Starting from the sixth week after performing piezocision, LLLT was carried out by the same operator (M.M.M). Gallium Aluminum Arsenide (Ga-Al-As) semiconductor diode laser (Klas^®^-DX Laser, Konftec Corporation, New Taipei City, Taiwan) was used with the following parameters: a wavelength of 808 nm, a power of 1.1 watts, and an energy of 4 joules/point with an application time of 15 s per point. The tip of the biostimulation handpiece was held in contact with the oral mucosa and perpendicular to the root axis during the laser procedure. A total of 32 irradiation points were performed each time, 16 on the buccal aspect and 16 on the palatal one, distributed as follows, with two irradiation points on the apical and cervical root of each of the six upper anterior teeth from both buccal and palatal aspects, so that the total application time was 60 s for one tooth. In addition, there were two irradiation points in the extraction space of the upper 1st premolar on both sides from both buccal and palatal aspects. After the initial application, the laser was applied again on days 3, 7, and 14, and then every 15 days up to 4 sessions. The en masse retraction phase was started 3 days after the piezocision procedure in the FC and FC+LLLT.

#### 2.5.4. En Masse Retraction Phase

The en masse retraction was achieved by using the sliding technique on the base archwires (0.019 3 0.025-in SS) using NiTi closed-coil springs (Ormco Corp, Orange, CA, USA). These springs were suspended from the soldered hooks distally to the lateral incisors to the TADs by applying a force of 250 g per side verified with a force gauge. Every two weeks, the force was checked and adjusted as necessary. The retraction was stopped when a Class I canine relation was achieved and a good incisor relationship was obtained, or spaces lateral to canines were closed.

### 2.6. Pain, Discomfort, Functional Impairments, and Outcome Measures

**Figure 4:**
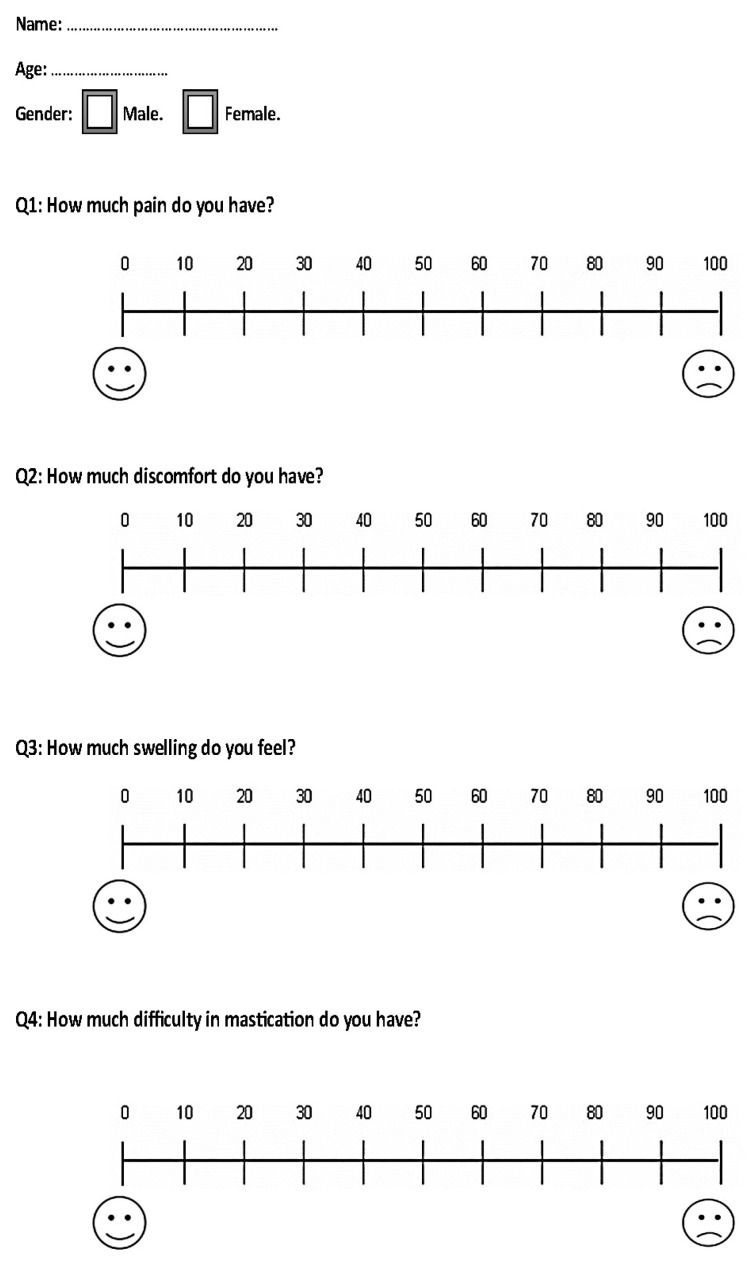
The questionnaire provided to the included patients at all assessment times except for the last assessment time, i.e., at 14 days after the activation of coil springs in the third month.

The study included two questionnaires distributed to patients within the first month of initiating en masse retraction. The first questionnaire was given to the patients at the following assessment times: 12 h (T1), one day (T2), 3 days (T3), 7 days (T4), and 14 days (T5) after the initiation of the en masse retraction. Additionally, during the third month, the same questionnaire was given at the following time intervals: 12 h (T6), 1 day (T7), 3 days (T8), 7 days (T9), and 14 days (T10) after the activation of coil springs. These questionnaires consisted of four questions regarding the patients’ experiences of pain, discomfort, and their feelings of swelling sensation and difficulty in mastication (Figure 4).

Based on the VAS scale, the severity of each studied variable was classified into the following categories: mild (less than 20), mild-to-moderate (from 20 to less than 40), moderate (from 40 to less than 60), moderate-to-severe (from 60 to less than 80), and severe (greater than 80) [25].

**Figure 5:**
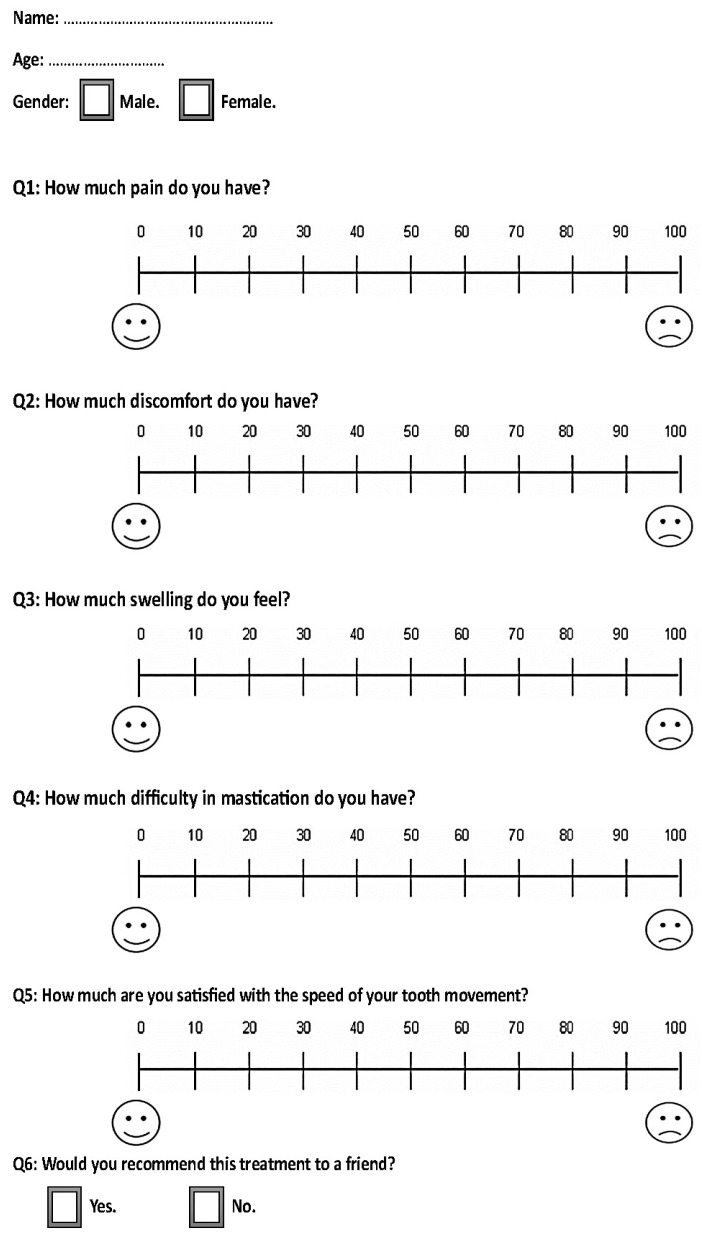
The questionnaire provided to the included patients at the last assessment time, i.e., at 14 days after the activation of coil springs in the third month.

The second questionnaire was given to the patients on the 14th day of the third month after retraction initiation (T10). It included the same questions as the first questionnaire, with the addition of two more questions: satisfaction levels assessed on a visual analog scale (VAS) and the possibility of recommending the same treatment to a friend (dichotomous scale) as another measure of satisfaction (Figure 5).

The questionnaire questions were explained in simple language to ensure patient understanding, and the researcher answered any questions without influencing the patient’s responses. The patients themselves answered the questions, selecting the option that best suited their experience. In cases of severe pain, patients were permitted to take Paracetamol 500 mg, provided that they took the painkiller following the completion of the questionnaire in order not the accuracy of their response.

## 3. Statistical Analysis

SPSS^®^ Version 20 (SPSS for Windows, version 20, IBM Corporation, New York, NY, USA) was the chosen software for statistical analysis. The Shapiro–Wilk test was used to distinguish the normal distribution of data. One-way ANOVA test or its alternative nonparametric test (i.e., Kruskal–Wallis test) was utilized to make a comparison between the three groups. For pairwise comparisons, the post hoc Bonferroni, LSD, or Games–Howell tests, or the latter’s alternative nonparametric test (i.e., Mann–Whitney test) was applied. Bonferroni’s correction of the significance level was applied due to the multiplicity of pairwise comparisons, and the results of all tests were considered significant at *p* ≤ 0.017.

## 4. Results

### 4.1. Baseline Sample Characteristics

The sample consisted of 60 patients (41 females and 19 males), with a mean age of 21.58 ± 3.19. There were no patient dropouts during the trial, and all patients in the three groups completed their questionnaires at all assessment time points. The baseline sample characteristics regarding age and gender distribution are given in Table 1.

### 4.2. Pain and Discomfort Levels

The greatest pain levels were reported 24 h after the initiation of the retraction during the first month in the three groups. The mean pain levels were mild to moderate in the FC+LLLT and FC groups, with 31.55 ± 7.09 mm and 33.7 ± 7.94 mm, respectively, while it was mild in the control group, with a mean pain level of 21.15 ± 7.8.

In the third month, the greatest pain levels were reported after 24 h (T7) in the three groups. The levels were mild in all three groups, with mean values of 9.10 ± 5.64 mm, 22.55 ± 7.39 mm, and 20.8 ± 7.13 mm in the FC+LLLT, FC, and control groups, respectively. In the 1st and 3rd months, the pain levels gradually decreased and became equal or close to zero after 14 days of assessment in all three groups, as seen in Table 2.

Similarly, the discomfort mean values were greatest after 24 h in the first and third months, and gradually decreased thereafter, becoming close to zero after 14 days, as shown in Table 3.

The pain levels were significantly smaller in the CONT group compared to both the FC and FC+LLLT groups at T1, T2, and T3; *p* < 0.001. However, the pain levels were significantly smaller in the FC+LLLT group compared to the CONT group and FC group at T6, T7, and T8, *p* < 0.001, as seen in Table 4. The discomfort levels were smaller in the CONT group compared to the FC+LLLT group and FC group at T1, T2, and T3, *p* < 0.001, as seen in Table 4.

### 4.3. Swelling Sensation

The greatest mean levels of swelling were observed after 24 h (T2) in the 1st month of retraction in the CONT, FC+LLLT, and FC groups 16.65 ± 8.41 mm, 39.65 ± 13.03 mm, and 38.3 ± 10.52 mm, respectively, as seen in Table 5. The patients in the CONT group reported significantly smaller levels of perceived swelling compared to the FC+LLLT, and FC groups at T1, T2, and T3 during the first month of the retraction (*p* ≤ 0.001; Table 5).

### 4.4. Chewing Difficulties

During the first month of assessment, the greatest mean levels of chewing difficulties were recorded at T2 and were generally ‘moderate’ in the FC+LLLT and FC groups; 40.45 ± 9.24, 42.75 ± 10.25, respectively, while they were in the ‘mild to moderate’ category at T2 in the CONT group; 24.8 ± 10.13.

In the third month of assessment, the greatest mean levels of chewing difficulties were at T7 in the three groups, and were classified as being ‘mild’ in FC+LLLT; 9.8 ± 4.22, ‘mild to moderate’ in FC and CONT groups (i.e., 19.6 ± 8.58, 19.1 ± 6.5, respectively). Then, the mean chewing difficulty values gradually decreased over time in the three groups until they approached zero or near zero at T10; Table 6.

The levels of chewing difficulties were significantly smaller in the CONT group at T1, T2, and T3 compared with the FC+LLLT and FC groups (*p* < 0.001). However, they were significantly smaller in the FC+LLLT group compared to FC and CONT groups at T6, T7, and T8 (*p* ≤ 0.001; Table 7).

### 4.5. Satisfaction with the Orthodontic Tooth Movement (OTM) Speed

The average time required for the en masse retraction phase was 7.85 months in the control group, 5.35 months in the FC group, and 4.5 months in the FC+LLLT group. Notably, the retraction period was significantly shorter in the FC+LLLT group compared to both the FC group (*p* = 0.009) and the control group (*p* < 0.001). Furthermore, the retraction period was also significantly shorter in the FC group when compared to the control group (*p* < 0.001).

High satisfaction levels with the OTM speed were reported in the FC+LLLT and FC groups, whereas levels of satisfaction were classified as being ‘moderate to high’ in the CONT group (i.e., mean VAS value: 84.95 mm, 80.3 and 77.25 mm, respectively, as seen in Table 6). Additionally, satisfaction with the OTM speed was greater in the FC+LLLT group compared with the FC and CONT groups (*p* < 0.05; Table 7). Furthermore, the majority of patients in the FC+LLLT and FC groups revealed that they would recommend their friends to have the same procedure, 95% and 90%, respectively, and would agree to undergo the same treatment again.

## 5. Discussion

The current study appears to be the first randomized clinical trial that evaluates the patient-reported outcome measures when using the combined piezocision-LLLT-assisted en masse retraction of upper anterior teeth in comparison with the precision-only-assisted retraction and the traditional method. Various surgical techniques have been developed to expedite dental movement, primarily relying on cortical cutting. These include traditional corticotomy [28], vestibular incision subperiosteal tunnel access [29], and flapless corticotomy using piezosurgery [30]. Among these, piezocision was selected for our study because it is considered the least aggressive among the methods mentioned. Surgical procedures for accelerating orthodontic tooth movement can be adopted in daily orthodontic treatment, provided they do not put an additional burden on patients, such as pain, discomfort, or functional impairments [25].

The VAS was used in this study for pain assessment. Despite its widespread acceptance in clinical research, it is crucial to note that the VAS is inherently subjective. This subjectivity is rooted in the fact that pain is a deeply personal experience. As such, the VAS scores reported in this study reflect the participants’ individual perception of their pain. This could potentially introduce a degree of variability into our results. However, despite this potential limitation, the VAS continues to be a valuable tool in research due to its simplicity and adaptability.

The patient-reported outcome measures were evaluated in the first and third months of retraction. The piezocision was only applied in the first month for both experimental groups, whereas the LLLT was only applied in the FC+LLLT experimental group in the third month.

### 5.1. The First Month of Assessment

Given that piezocision was applied in both experimental groups before retraction, the comparisons made are confined to the first month only regarding studies that employed piezocision-assisted orthodontic tooth movement. The pain and discomfort levels were in the ‘mild to moderate’ category after 24 h of retraction in the three groups. However, after 72 h of retraction, they were classified as ‘mild to moderate’ in the experimental groups, and ‘mild’ in the control group. Additionally, the levels of pain and discomfort were significantly greater in FC+LLLT and FC groups compared to the control group after 12, 24, and 72 h (*p* < 0.001). The greater pain levels in both FC+LLLT and FC groups may be attributed to the surgical procedure applied to the patients of these groups compared to the control group.

The current results were inconsistent with those of the study of Hatrom et al. [31], in which the pain levels were mild in the piezocision and control groups during the first 24 h after the initiation of retraction. This discrepancy may be attributed to the fact that in the current trial, incisions were made on both the buccal and palatal sides of the alveolar bone, whereas in Hatrom’s study, the incisions were only placed buccally.

The results of this study disagreed with those reported by Alfawal et al., who evaluated the patient-reported outcome measures associated with canine retraction accelerated by piezosurgery or laser-assisted flapless corticotomy in a compound design RCT. Alfawal et al. found that the levels of pain were moderate and the levels of discomfort were severe in the piezocision group after 24 h of canine retraction in the Alfawal et al. study [32]. This inconsistency may be due to the difference in the onset of retraction force, which was on the same day of the surgical intervention in the study of Alfawal et al. [32], whereas it was four days postoperatively in the current work. The study of Alfawal et al. had a compound design, i.e., two parallel groups with a split-mouth design in each group. This kind of study design may distort patient perception of pain that may originate only from one side of the mouth. Additionally, the assessment of pain on one side of the mouth does not provide a whole picture of the pain that would be encountered when the procedure is performed bilaterally [25].

Patient perception of swelling was ‘mild to moderate’ in the experimental groups and ‘mild’ in the control group after 12, 24, and 72 h of retraction. This can be attributed to edema caused by the surgical procedure in both the FC+LLLT and FC groups. This is consistent with a study by Gibreal et al., which also found increased perception of swelling following piezocision-assisted leveling and alignment [27].

After 24 h of retraction commencement, patients in the experimental groups reported ‘moderate’ levels of chewing difficulty, while those in the control group reported ‘mild to moderate’ levels. Then, after 72 h, the levels of chewing difficulties decreased to the ‘mild to moderate’ category in the experimental groups and were classified as mild in the control group. This can be attributed to the pain and swelling caused by the surgical procedure in the FC+LLLT and FC groups. These findings are consistent with those reported by Alfawal et al. and Gibreal et al. [27,33].

### 5.2. The Third Month of Assessment

In the third month of retraction, frequent LLLT application was continued in the FC+LLLT group, while the FC and CONT groups did not undergo any further intervention. The primary aim of the laser application was to accelerate orthodontic tooth movement, as previous reports have demonstrated that laser irradiation can accelerate the en masse retraction of upper anterior teeth by 26–54% [34,35,36]. However, in this study, the focus was on patient-centered outcomes during the acceleration of orthodontic tooth movement, with the application of laser from the sixth week following retraction initiation. Therefore, comparisons in the third month were made with other studies that have utilized LLLT-assisted acceleration.

After 12, 24, and 72 h of re-activating the coil springs for the en masse retraction, patients in the FC+LLLT group experienced lower levels of pain and discomfort compared to the FC and CONT groups. After 24 h, the levels were classified as ‘mild to moderate’ in the FC and CONT groups, while they were classified as ‘mild’ in the FC+LLLT group. These lower levels in the FC+LLLT group can be attributed to the dual mechanism of low-level laser therapy, which targets both local and systemic aspects of pain [37], in addition to its role in accelerating orthodontic tooth movement.

The results of this study agreed with those reported by Bhat et al., who found that the LLLT was effective in relieving pain when retracting upper anterior teeth [20]. However, the pain level was assessed only at one time point, which was 7 days after laser application, when most of the pain resulting from orthodontic treatment had already subsided spontaneously [38].

On the other hand, the results of this study disagreed with those of the study of Dalaie et al., who studied the effects of LLLT on canine retraction in a split-mouth-designed RCT. They found that the pain levels were classified as ‘mild to moderate’ after 24 h of retraction in both the experimental and control groups, with no differences between them [39]. This discrepancy can be explained by the differences in laser irradiation parameters. In their study, the retracted canines in the LLLT group were irradiated with an 880 nm Ga-Al-As laser at a dosage of 5 j/cm2 for 10 s at 8 spots, while in the current study, a similar device was used but with a dosage of 4 joules/point for 15 s per point at 32 spots.

The levels of chewing difficulty were classified as ‘mild’ in the FC+LLLT group, whereas they were ‘mild to moderate’ in the FC and CONT groups at 24 h following force application in the third month. This can be explained by the effect of LLLT in the FC+LLLT group, which resulted in reduced levels of pain, and, therefore, reduced chewing difficulties.

The assessment of patient satisfaction in OTM speed with the applied acceleratory procedures revealed a high level of satisfaction in both experimental groups, but the mean scores were greater in the FC+LLLT group than the FC group, and this could be explained by the additional effect of LLLT during the third month of treatment. The finding of high satisfaction was similar to what was observed by Alfawal et al., who reported a mean VAS satisfaction score of 82.94 mm. Additionally, there was an agreement with the study of Khlef et al., which evaluated the patient-reported outcome measures associated with piezocision-assisted en masse retraction of upper anterior teeth, and found that most patients in the piezocision group would recommend the accelerated treatment modality to their friends [40].

## 6. Limitations

Due to the nature of the study, one of the limitations was the impossibility of blinding the researcher or the patients. Therefore, only the outcome assessor was subject to blinding. Additionally, our study potentially faces the limitation of the inherent subjectivity of the visual analog scale (VAS), which relies on an individual’s personal perception of their pain. This subjectivity could introduce a degree of variability into our findings [41]. Moreover, the absence of daily assessments during the first postoperative week may have limited understanding of the immediate impact of the procedure on pain, discomfort, and functional impairment. It is essential for future studies to investigate the potential long-term complications arising from corticotomy and LLLT interventions, including root resorption, scars, and the vitality of teeth. Furthermore, the evaluation of periodontal indices such as gingival recession and alveolar bone levels after the treatment requires further attention. Lastly, it is recommended to assess patient-centered outcomes when using alternative acceleration methods during orthodontic treatment.

## 7. Conclusions

The study found that piezocision-accelerated retraction caused mild to moderate discomfort, swelling, and chewing difficulties on the first day, which were significantly higher than in the control group. However, the addition of low-level laser therapy (LLLT) significantly reduced these sensations compared to both the piezocision-only and control groups. Overall, the combination of piezocision and LLLT proved highly effective in reducing pain, discomfort, and chewing difficulties and enhancing patient satisfaction compared to conventional methods. Thus, LLLT is a valuable adjunct to piezocision, improving both patient experience and outcomes.

## Figures and Tables

**Table 1:** Basic sample characteristics (gender and age).

		FC+LLLT	FC	CONT	Overall
Gender	Male *n* (%)	4 (20%)	5 (25%)	5 (25%)	14 (23.3%)
	Female *n* (%)	16 (80%)	15 (75%)	15 (75%)	46 (76.7%)
Age	Mean ± SD	21.65 ± 2.98	21.00 ± 3.28	22.1 ± 3.19	21.58 ± 3.19

FC+LLLT: flapless corticotomy combined with the later application of LLLT group, FC: flapless corticotomy group, CONT: control group.

**Table 2:** Descriptive statistics of pain intensity scores (in mm) at ten assessment times in the three groups using visual analog scales and the results of the significance testing.

		Group	Mean (SD)	Median	Range (0–100)	*p*-Value *
1st month	T1 ^a^	FC+LLLT	19.15 (3.94)	18.5	12–26	<0.001
		FC	19.25 (4.15)	18.5	13–30	
		CONT	13.25 (3.68)	12	8–22	
	T2 ^b^	FC+LLLT	31.55 (7.09)	29	22–48	<0.001
		FC	33.70 (7.94)	34	20–50	
		CONT	21.15 (7.80)	20	10–35	
	T3 ^b^	FC+LLLT	20.75 (6.49)	19	12–34	<0.001
		FC	21.50 (5.12)	20.5	10–30	
		CONT	13.45 (6.34)	15	1–25	
	T4 ^b^	FC+LLLT	6.10 (3.19)	5.5	2–13	0.597
		FC	5.55 (3.72)	5	1–13	
		CONT	5.00 (3.29)	5	0–10	
	T5 ^a^	FC+LLLT	1.00 (0.86)	1	0–3	0.719
		FC	0.95 (0.69)	1	0–2	
		CONT	0.80 (0.77)	1	0–2	
3rd month	T6 ^a^	FC+LLLT	5.10 (3.02)	5	1–10	<0.001
		FC	12.05 (5.06)	11	1–22	
		CONT	11.55 (2.28)	11	8–18	
	T7 ^b^	FC+LLLT	9.10 (5.64)	10	2–21	<0.001
		FC	22.55 (7.39)	21.5	10–38	
		CONT	20.80 (7.13)	20	10–33	
	T8 ^b^	FC+LLLT	6.25 (2.86)	6	1–11	<0.001
		FC	14.90 (7.05)	12	5–15	
		CONT	14.40 (7.74)	12.5	5–30	
	T9 ^a^	FC+LLLT	1.70 (1.53)	1	0–5	0.006
		FC	5.50 (5.24)	5	1–20	
		CONT	4.30 (2.99)	5	0–9	
	T10 ^a^	FC+LLLT	0.00 (0.00)	0	0	0.018
		FC	1.40 (2.48)	0	0–8	
		CONT	1.10 (1.65)	0	0–5	

SD: standard deviation; Min: minimum; Max: maximum; 1st month’s assessment times—T1: after 12 h of the beginning of en masse retraction; T2: after 24 h; T3: after 3 days; T4: after 7 days; T5: after 14 days; 3rd month’s assessment times—T6: after 12 h; T7: after 24 h; T8: after 3 days; T9: after 7 days; T10: after 14 days. FC+LLLT: flapless corticotomy combined with the later application of LLLT group, FC: flapless corticotomy group, CONT: control group. ^a^: One-way ANOVA, ^b^: Kruskal–Wallis. * Significant at the 0.005 level with Bonferroni’s adjustment of the level of significance.

**Table 3:** Descriptive statistics discomfort score (in mm) at ten assessment times in the three groups using visual analog scales and the results of the significance testing.

		Group	Mean (SD)	Median	Range (0–100)	*p*-Value *
1st month	T1 ^a^	FC+LLLT	29.95 (9.54)	30	18–50	<0.001
		FC	31.45 (13.56)	31.5	15–55	
		CONT	17.35 (6.11)	16.5	9–30	
	T2 ^a^	FC+LLLT	38.50 (10.17)	40	22–60	<0.001
		FC	39.35 (13.64)	41	15–62	
		CONT	24.50 (6.22)	25	15–38	
	T3 ^a^	FC+LLLT	22.85 (10.43)	19.5	8–47	<0.001
		FC	24 (9.64)	18.5	4–39	
		CONT	12.55 (6.57)	12.5	3–25	
	T4 ^b^	FC+LLLT	7.90 (5.54)	7	1–18	0.441
		FC	8.50 (4.97)	8.5	2–24	
		CONT	6.35 (4.43)	5	0–15	
	T5 ^b^	FC+LLLT	3.60 (3.39)	2	1–12	0.030
		FC	3.90 (3.26)	4	0–12	
		CONT	2.00 (2.75)	0	0–8	
3rd month	T6 ^a^	FC+LLLT	11.70 (8.40)	10.5	1–25	0.023
		FC	19.25 (10.42)	19	5–36	
		CONT	17.40 (7.16)	17	4–30	
	T7 ^a^	FC+LLLT	16.00 (10.46)	16.5	1–33	0.075
		FC	22.90 (9.95)	20	10–42	
		CONT	21.10 (8.63)	22.5	5–36	
	T8 ^b^	FC+LLLT	9.05 (6.61)	8	1–20	0.110
		FC	14.65 (9.82)	15	1–33	
		CONT	12.55 (6.57)	12.5	3–25	
	T9 ^b^	FC+LLLT	3.95 (3.39)	2.5	1–11	0.437
		FC	5.40 (4.10)	5	1–15	
		CONT	5.65 (4.39)	5	0–15	
	T10 ^b^	FC+LLLT	1.00 (0.00)	1	1–1	0.021
		FC	1.00 (0.00)	1	1–1	
		CONT	0.95 (1.50)	0	0–5	

SD: standard deviation; Min: minimum; Max: maximum; 1st month’s assessment times—T1: after 12 h of the beginning of en masse retraction; T2: after 24 h; T3: after 3 days; T4: after 7 days; T5: after 14 days; 3rd month’s assessment times—T6: after 12 h; T7: after 24 h; T8: after 3 days; T9: after 7 days; T10: after 14 days. FC+LLLT: flapless corticotomy combined with the later application of LLLT group, FC: flapless corticotomy group, CONT: control group. ^a^: One-way ANOVA, ^b^: Kruskal–Wallis. * Significant at the 0.005 level with Bonferroni’s adjustment of the level of significance.

**Table 4:** Post hoc tests for pairwise comparisons concerning questions 1 and 2 of the questionnaires.

			Groups	Mean Difference	95% CI for Difference	*p*-Value *
					Lower, Upper Boundaries	
Q1: Pain levels	1st month	T1 ^a^	CONT–FC	−6	−9.07, −2.94	<0.001
			CONT–FC+LLLT	−5.9	−8.97, −2.84	<0.001
			FC–FC+LLLT	0.1	−2.97, 3.17	0.957
		T2 ^b^	CONT–FC	−14.55	−17.37, −7.73	<0.001
			CONT–FC+LLLT	−12.40	−15.22, −5.58	<0.001
			FC–FC+LLLT	2.15	−2.67, 6.97	0.376
		T3 ^b^	CONT–FC	−8.05	−11.86, −4.24	<0.001
			CONT–FC+LLLT	−7.30	−11.11, −3.49	<0.001
			FC–FC+LLLT	0.75	−3.06, 4.56	0.695
	3rd month	T6 ^a^	CONT–FC	−0.5	−3.35, 2.35	0.902
			CONT–FC+LLLT	6.45	3.60, 9.30	<0.001
			FC–FC+LLLT	6.95	4.10, 9.80	<0.001
		T7 ^b^	CONT–FC	−1.75	−6.03, 2.53	0.417
			CONT–FC+LLLT	10.70	6.42, 14.98	<0.001
			FC–FC+LLLT	12.45	8.17, 16.73	<0.001
		T8 ^c^	CONT–FC	−0.50	−6.21, 5.21	0.975
			CONT–FC+LLLT	8.15	3.54, 12.76	0.001
			FC–FC+LLLT	8.65	4.41, 12.89	<0.001
Q2: Discomfort levels	1st month	T1 ^b^	CONT–FC	−14.10	−22.36, −5.84	0.001
			CONT–FC+LLLT	−12.60	−18.82, −6.38	<0.001
			FC–FC+LLLT	1.50	−7.58, 10.58	0.914
		T2 ^b^	CONT–FC	−14.85	−23.17, −6.53	<0.001
			CONT–FC+LLLT	−14.00	−20.56, −7.44	<0.001
			FC–FC+LLLT	0.85	−8.46, 10.16	0.937
		T3 ^b^	CONT–FC	−11.45	−17.85, −5.04	<0.001
			CONT–FC+LLLT	−10.3	−17.07, −3.52	0.002
			FC–FC+LLLT	1.15	−6.60, 8.90	0.931

SD: standard deviation; Min: minimum; Max: maximum; 1st month’s assessment times—T1: after 12 h of the beginning of en masse retraction; T2: after 24 h; T3: after 3 days; T4: after 7 days; T5: after 14 days; 3rd month’s assessment times—T6: after 12 h; T7: after 24 h; T8: after 3 days; T9: after 7 days; T10: after 14 days. FC+LLLT: flapless corticotomy combined with the later application of LLLT group, FC: flapless corticotomy group, CONT: control group. ^a^: Mann–Whitney U, ^b^: LSD, ^c^: Games–Howell. * significant at the 0.017 level.

**Table 5:** Descriptive statistics swelling sensation score (in mm) at ten assessment times in the three groups using visual analog scales and the results of the significance testing.

		Group	Mean (SD)	Median	Range (0–100)	*p*-Value *
1st month	T1 ^a^	FC+LLLT	28.60 (10.00)	28.5	10–55	<0.001
		FC	26.10 (14.25)	22	10–65	
		CONT	8.25 (5.18)	9	0–18	
	T2 ^b^	FC+LLLT	39.65 (13.03)	39.5	15–60	<0.001
		FC	38.30 (10.52)	38.5	25–62	
		CONT	16.65 (8.41)	18.5	1–29	
	T3 ^b^	FC+LLLT	29.55 (17.64)	26	6–71	<0.001
		FC	31.30 (17.84)	31	5–58	
		CONT	6.55 (4.15)	6	1–15	
	T4 ^a^	FC+LLLT	3.20 (2.88)	2	0–10	0.077
		FC	3.80 (2.80)	5	0–10	
		CONT	2.15 (2.41)	1	0–9	
	T5 ^a^	FC+LLLT	1.35 (1.57)	1	0–5	0.148
		FC	1.75 (1.65)	1	0–7	
		CONT	1.10 (1.45)	0.5	0–5	
3rd month	T6 ^a^	FC+LLLT	2.85 (2.58)	1	1–10	0.198
		FC	4.50 (3.59)	5	1–15	
		CONT	4.00 (3.28)	3	0–12	
	T7 ^a^	FC+LLLT	3.45 (2.95)	3	1–12	0.019
		FC	6.85 (4.57)	5	1–18	
		CONT	5.55 (3.72)	5	0–15	
	T8 ^a^	FC+LLLT	2.00 (2.64)	1	1–11	0.007
		FC	4.30 (2.74)	4	1–10	
		CONT	3.30 (3.28)	2	0–10	
	T9 ^a^	FC+LLLT	1.05 (0.22)	1	1–2	0.466
		FC	1.00 (0.00)	1	1–1	
		CONT	1.10 (1.17)	1	0–5	
	T10 ^a^	FC+LLLT	0.20 (0.41)	0	0–1	0.680
		FC	0.15 (0.37)	0	0–1	
		CONT	0.10 (0.31)	0	0–1	

SD: standard deviation; Min: minimum; Max: maximum; 1st month’s assessment times—T1: after 12 h of the beginning of en masse retraction; T2: after 24 h; T3: after 3 days; T4: after 7 days; T5: after 14 days; 3rd month’s assessment times—T6: after 12 h; T7: after 24 h; T8: after 3 days; T9: after 7 days; T10: after 14 days. FC+LLLT: Flapless corticotomy combined with the later application of LLLT group, FC: Flapless corticotomy group, CONT: Control group. ^a^: Kruskal-Wallis, ^b^: One-way ANOVA. * Significant at the 0.005 level with Bonferroni’s adjustment of the level of significance.

**Table 6:** Descriptive statistics of questions 4 and 5 score (in mm) at ten assessment times in the three groups using visual analog scales and the results of the significance testing.

			Group	Mean (SD)	Median	Range (0–100)	*p*-Value
Q4: Chewing difficulty levels	1st month	T1 ^a^	FC+LLLT	29.50 (6.67)	29.5	20–40	<0.001 *
			FC	31.55 (6.33)	30	20–40	
			CONT	16.30 (8.61)	15	4–30	
		T2 ^a^	FC+LLLT	40.45 (9.24)	39	25–60	<0.001 *
			FC	42.75 (10.25)	38.5	30–65	
			CONT	24.80 (10.13)	24.5	10–48	
		T3 ^b^	FC+LLLT	25.50 (13.03)	23.5	7–50	<0.001 *
			FC	27.10 (11.92)	22	14–48	
			CONT	6.15 (4.59)	5.5	1–15	
		T4 ^b^	FC+LLLT	4.50 (3.27)	5	1–10	0.077
			FC	5.50 (4.21)	5	1–15	
			CONT	2.85 (3.00)	2	0–9	
		T5 ^b^	FC+LLLT	1.40 (1.73)	1	0–5	0.610
			FC	1.55 (2.04)	1	0–8	
			CONT	1.05 (1.47)	0.5	0–5	
	3rd month	T6 ^b^	FC+LLLT	3.80 (2.97)	3	1–10	<0.001 *
			FC	8.80 (4.34)	11.5	1–18	
			CONT	9.6 (6.5)	10	1–15	
		T7 ^a^	FC+LLLT	9.8 (4.22)	10.5	3–18	<0.001 *
			FC	19.60 (8.58)	18	8–40	
			CONT	19.10 (6.50)	19.5	10–35	
		T8 ^b^	FC+LLLT	2.95 (3.43)	1	1–12	0.001 *
			FC	7.75 (3.64)	7	1–15	
			CONT	6.65 (5.82)	5	1–20	
		T9 ^b^	FC+LLLT	1.00 (0.00)	1	1–1	0.012
			FC	2.75 (2.90)	1	1–10	
			CONT	3.10 (3.31)	1	1–10	
		T10 ^b^	FC+LLLT	0.40 (0.50)	0	0–1	0.806
			FC	0.30 (0.47)	0	0–1	
			CONT	0.35 (0.49)	0	0–1	
Q5: Satisfaction with OTM speed		T10 ^a^	FC+LLLT	88.65 (7.44)	89	100–70	<0.001 **
			FC	80.3 (8.96)	79.5	92–59	
			CONT	72.25 (7.14)	73	85–58	

SD: standard deviation; Min: minimum; Max: maximum; 1st month’s assessment times—T1: after 12 h of the beginning of en masse retraction; T2: after 24 h; T3: after 3 days; T4: after 7 days; T5: after 14 days; 3rd month’s assessment times—T6: after 12 h; T7: after 24 h; T8: after 3 days; T9: after 7 days; T10: after 14 days. OTM: orthodontic tooth movement, FC+LLLT: flapless corticotomy combined with the later application of LLLT group, FC: flapless corticotomy group, CONT: control group. ^a^: One-way ANOVA, ^b^: Kruskal–Wallis. * significant at the 0.005 level With Bonferroni Adjustment. ** significant at the 0.017 level with Bonferroni’s adjustment.

**Table 7:** Post hoc tests for pairwise comparisons concerning questions 3, 4, and 5 of the questionnaires.

			Groups	Mean Difference	95% CI for Difference	*p*-Value *
					Lower, Upper Boundaries	
Q3: Swelling sensation levels	1st month	T1 ^a^	CONT–FC	−17.85	−26.03, −9.67	<0.001
			CONT–FC+LLLT	−20.35	−28.53, −12.17	<0.001
			FC–FC+LLLT	−2.5	−10.68, 5.68	0.250
		T2 ^b^	CONT–FC	−21.65	−28.50, −14.80	<0.001
			CONT–FC+LLLT	−23.00	−29.85, −16.15	<0.001
			FC–FC+LLLT	−1.35	−8.20, 5.50	0.695
	3rd month	T6 ^c^	CONT–FC	−24.75	−35.07, −14.43	<0.001
			CONT–FC+LLLT	−23.00	−33.21, −12.79	<0.001
			FC–FC+LLLT	1.75	−11.93, 15.43	0.948
Q4: Chewing difficulty levels	1st month	T1 ^b^	CONT–FC	−15.25	−19.85, −10.65	<0.001
			CONT–FC+LLLT	−13.20	−17.80, −8.60	<0.001
			FC–FC+LLLT	2.05	−2.55, 6.65	0.376
		T2 ^b^	CONT–FC	−17.95	−24.21, −11.69	<0.001
			CONT–FC+LLLT	−15.65	−21.91, −9.39	<0.001
			FC–FC+LLLT	2.30	−3.96, 8.56	0.465
		T3 ^a^	CONT–FC	−20.95	−29.17, −12.73	<0.001
			CONT–FC+LLLT	−19.35	−27.57, −11.13	<0.001
			FC–FC+LLLT	1.6	−6.62, 9.82	0.655
	3rd month	T6 ^a^	CONT–FC	−1.6	−5.71, 4.71	0.486
			CONT–FC+LLLT	3.4	3.34, 13.76	<0.001
			FC–FC+LLLT	5	3.84, 14.26	<0.001
		T7 ^c^	CONT–FC	−0.50	−6.39, 5.39	0.977
			CONT–FC+LLLT	9.3	4.29, 12.81	<0.001
			FC–FC+LLLT	9.05	3.76, 14.34	0.001
		T8 ^a^	CONT–FC	−1.1	−1.63, 2.33	0.168
			CONT–FC+LLLT	3.7	0.12, 4.08	0.015
			FC–FC+LLLT	4.8	−0.23, 3.73	<0.001
Q5: Satisfaction with OTM speed	3rd month	T10 ^d^	CONT–FC	−8.05	−14.22, −1.87	0.006
			CONT–FC+LLLT	−16.4	−10.24, −22.55	<0.001
			FC–FC+LLLT	−8.35	−14.5, −2.19	0.004

SD: standard deviation; Min: minimum; Max: maximum; 1st month’s assessment times—T1: after 12 h of the beginning of en masse retraction; T2: after 24 h; T3: after 3 days; T4: after 7 days; T5: after 14 days. 3rd month’s assessment times—T6: after 12 h; T7: after 24 h; T8: after 3 days; T9: after 7 days; T10: after 14 days. FC+LLLT: flapless corticotomy combined with the later application of LLLT group, FC: flapless corticotomy group, CONT: control group. ^a^: Mann–Whitney U, ^b^: LSD, ^c^: Games–Howell, ^d^: Bonferroni. * significant at the 0.017 level.

## Data Availability

Datasets and spreadsheets detailing the current work are available upon reasonable request from the corresponding authors.
